# The deubiquitinating enzyme OTUD7b protects dendritic cells from TNF-induced apoptosis by stabilizing the E3 ligase TRAF2

**DOI:** 10.1038/s41419-023-06014-5

**Published:** 2023-07-29

**Authors:** Kunjan Harit, Rituparna Bhattacharjee, Kai Matuschewski, Jennifer Becker, Ulrich Kalinke, Dirk Schlüter, Gopala Nishanth

**Affiliations:** 1 Institute of Medical Microbiology and Hospital Epidemiology, Hannover Medical School, 30625 Hannover, Germany; 2 Department of Molecular Parasitology, Institute of Biology, Humboldt University, 10115 Berlin, Germany; 3 Institute for Experimental Infection Research, TWINCORE, Centre for Experimental and Clinical Infection Research, a joint venture between the Helmholtz Centre for Infection Research and the Hannover Medical School, Hannover, Germany

**Keywords:** Cell death and immune response, Immune cell death

## Abstract

The cytokine tumor necrosis factor (TNF) critically regulates the intertwined cell death and pro-inflammatory signaling pathways of dendritic cells (DCs) *via* ubiquitin modification of central effector molecules, but the intrinsic molecular switches deciding on either pathway are incompletely defined. Here, we uncover that the ovarian tumor deubiquitinating enzyme 7b (OTUD7b) prevents TNF-induced apoptosis of DCs in infection, resulting in efficient priming of pathogen-specific CD8^+^ T cells. Mechanistically, OTUD7b stabilizes the E3 ligase TNF-receptor-associated factor 2 (TRAF2) in human and murine DCs by counteracting its K48-ubiquitination and proteasomal degradation. TRAF2 in turn facilitates K63-linked polyubiquitination of RIPK1, which mediates activation of NF-κB and MAP kinases, IL-12 production, and expression of anti-apoptotic cFLIP and Bcl-xL. We show that mice with DC-specific OTUD7b-deficiency displayed DC apoptosis and a failure to induce CD8^+^ T cell-mediated brain pathology, experimental cerebral malaria, in a murine malaria infection model. Together, our data identify the deubiquitinating enzyme OTUD7b as a central molecular switch deciding on survival of human and murine DCs and provides a rationale to manipulate DC responses by targeting their ubiquitin network downstream of the TNF receptor pathway.

## Introduction

Dendritic cells (DCs) are professional antigen-presenting cells regulating both innate and adaptive immune responses [1–4]. The activation and function of DCs is strongly dependent on the nuclear factor “kappa light-chain enhancer” of activated B cells (NF-κB) pathway, which in turn is tightly regulated by the ubiquitin system [5, 6]. Ubiquitination is a central post-translational modification regulating numerous cellular functions, including innate and adaptive immune responses and cell survival [6–10]. Ubiquitination is catalyzed by ubiquitin (Ub)-activating (E1), -conjugating (E2) and -ligating (E3) enzymes [11]. A single Ub molecule can be attached to the target protein, or Ub chains can be formed through the conjugation of Ub molecules to one of the seven lysine residues (K6, K11, K27, K29, K33, K48 or K63) of another Ub molecule. Currently, the best characterized Ub functions are K11/K48-linked ub-mediated proteasomal degradation and K63-linked ub-mediated regulation of non-degradative functions of target proteins [8, 12–15]. Ubiquitination can be counteracted by deubiquitinating enzymes (DUBs) which specifically removes Ub molecules from the substrate [16].

The pleiotropic cytokine TNF plays an important role in the maturation and survival of both human and murine DCs [17–20]. Stimulation of TNF receptor-1 (TNFR1) by soluble TNF triggers the formation of TNFR1 complex-I comprising of the E3 ligase TNFR-associated-factor 2 (TRAF2), TNFR1-associated via death domain (TRADD), cellular inhibitor of apoptosis (c-IAP) 1/2 and receptor-interacting serine/threonine-protein kinase (RIPK) 1, which induces the activation of NF-κB and mitogen-activated protein (MAP) kinase pathways and expression of antiapoptotic molecules- cellular FLICE-like inhibitory protein (cFLIP) and B-cell lymphoma-extra large (Bcl-xL) [21] resulting in cell survival. Alternatively, TNF stimulation can result in the formation of a cytosolic complex comprising of either procaspase-8, TRADD and Fas-associated protein with death domain (FADD) (complex IIa), procaspase-8, RIPK1, and FADD (complex IIb) or RIPK1, RIPK3, MLKL (complex III) which may induce caspase-mediated apoptosis (complexes IIa and b) or MLKL-mediated necroptosis [22].

The TNF-induced pro-inflammatory, survival and cell death pathways are strongly regulated by the dynamic ubiquitination and deubiquitination of signaling molecules [23–25]. OTUD7b can cleave K11- [26], K48- [27] and K63- linked polyubiquitin chains from substrates [20], thereby negatively regulates activation of the canonical NF-κB pathway by removing K63-linked polyubiquitin chains from RIPK1 [28] and also noncanonical NF-κB signaling by cleaving K48-linked poly-ubiquitin chains from TRAF3 [27]. However, the function of OTUD7b in the regulation of DCs is currently unknown

To investigate the role of OTUD7b in DC activation, survival and induction of CD8^+^ T cell responses, we established a novel mouse strain with conditional deletion of OTUD7b in DCs and employed the model of *Plasmodium berghei* ANKA (*Pb*A)-induced experimental cerebral malaria (ECM), a mouse model of human cerebral malaria (CM). Both human CM and murine ECM are characterized by high levels of circulating TNF [29–31]. In CM, TNF is a double-edged sword: on the one hand TNF contributes to the control of the infection, on the other hand TNF may contribute to the expression of cell adhesion molecules on the endothelial cells leading to enhanced sequestration of infected RBCs and increased endothelial cell permeability, thereby fostering brain pathology [32].

The hallmark of ECM is the accumulation and adherence of pathogenic CD8^+^ T cells in cerebral blood vessels [33–35]. In ECM, conventional CD11c^+^ CD8α^+^ DCs process and present parasitic antigens to CD8^+^ T cells, which subsequently contribute to the disruption of the blood-brain barrier (BBB) through granzyme B and perforin-mediated cytotoxicity [36–39].

We show that upon infection with *Pb*A DC-specific OTUD7b prevents TNF-induced apoptosis and augments activation of the NF-κB and MAP kinase pathways. Mechanistically, OTUD7b stabilized TRAF2 by deconjugating K48-linked polyubiquitin chains, thereby, fostering activation of NF-κB and MAP kinase pathways and preventing apoptosis of DCs. The OTUD7b-dependent DC survival lead to the priming of pathogen-specific CD8^+^ T cells and, finally, lethal course of ECM. In good agreement, DC-specific ablation of OTUD7b induced apoptosis of DCs, prevented the induction of pathogenic CD8^+^ T cells and completely protected from ECM.

## Results

### DC-specific OTUD7b prevents DC death and protects mice from ECM

**Fig. 1:**
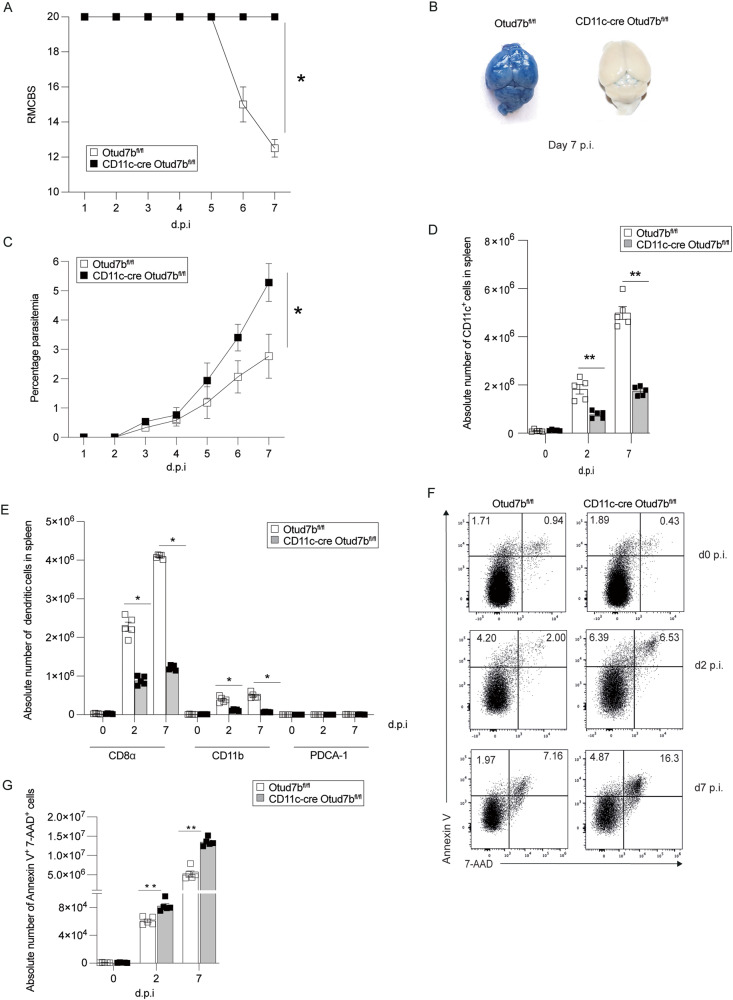
DC-specific deletion of OTUD7b induce DC death and confers protection against *Plasmodium berghei ANK (Pb*A*)*-induced experimental cerebral malaria. **A-I** Otud7b^fl/fl^ and CD11c-cre Otud7b^fl/fl^ mice were infected i.p. with 1×10^6^ *Pb*A-infected red blood cells (iRBCs). **A** Mice were monitored once per day for neurological signs according to RMCBS scale until day 7 p.i. A RMCBS score of 20 represents completely healthy mice. Data represent one of two independent experiments, (*n* = 10, *p < 0.05) (Mann–Whitney U test). **B** Representative image of Evans blue assay performed at day 7 p.i. Mice were injected i.v. with Evans blue dye and perfused with 0.9% saline 1 h post infection. Brains were isolated and photographed. Two independent experiments with 3 mice per experimental group and experiment were performed. (**C**) Peripheral blood parasitemia was measured daily during the course of infection by microscopy of Giemsa-stained thin blood smears (*n* = 5 per group, two independent experiments). **D** Absolute cell numbers of CD11c^+^ DCs in spleen, analyzed at days 0, 2 and 7 p.i. by flow cytometry (*n* = 5 per group). **E** Absolute cell numbers of CD11c^+^ CD8α^+^, CD11c^+^ CD11b^+^ and CD11c^+^ PDCA1^+^, respectively, DC subtypes at days 0, 2 and 7 p.i. (*n* = 5 per group). Flow cytometric analysis of DC death was performed using annexin V and 7AAD staining of splenic CD11c^+^ DCs at day 0 (uninfected), 2 and 7 p.i. **F** Representative flow cytometry dot plots of AnnexinV/7AAD stained CD11c^+^ DCs from spleen of Otud7b^fl/fl^ and CD11c-Cre Otud7b^fl/fl^ mice. Representative dot plots are shown (*n* = 5 per group). **G** Absolute number of AnnexinV/7AAD double positive CD11c^+^ dendritic cells from spleen of Otud7b^fl/fl^ and CD11c-Cre Otud7b^fl/fl^ mice (*n* = 5 per group). All bars represent mean values + SEM. Student’s t-test *p < 0.05, **p < 0.01.

**Fig. 2:**
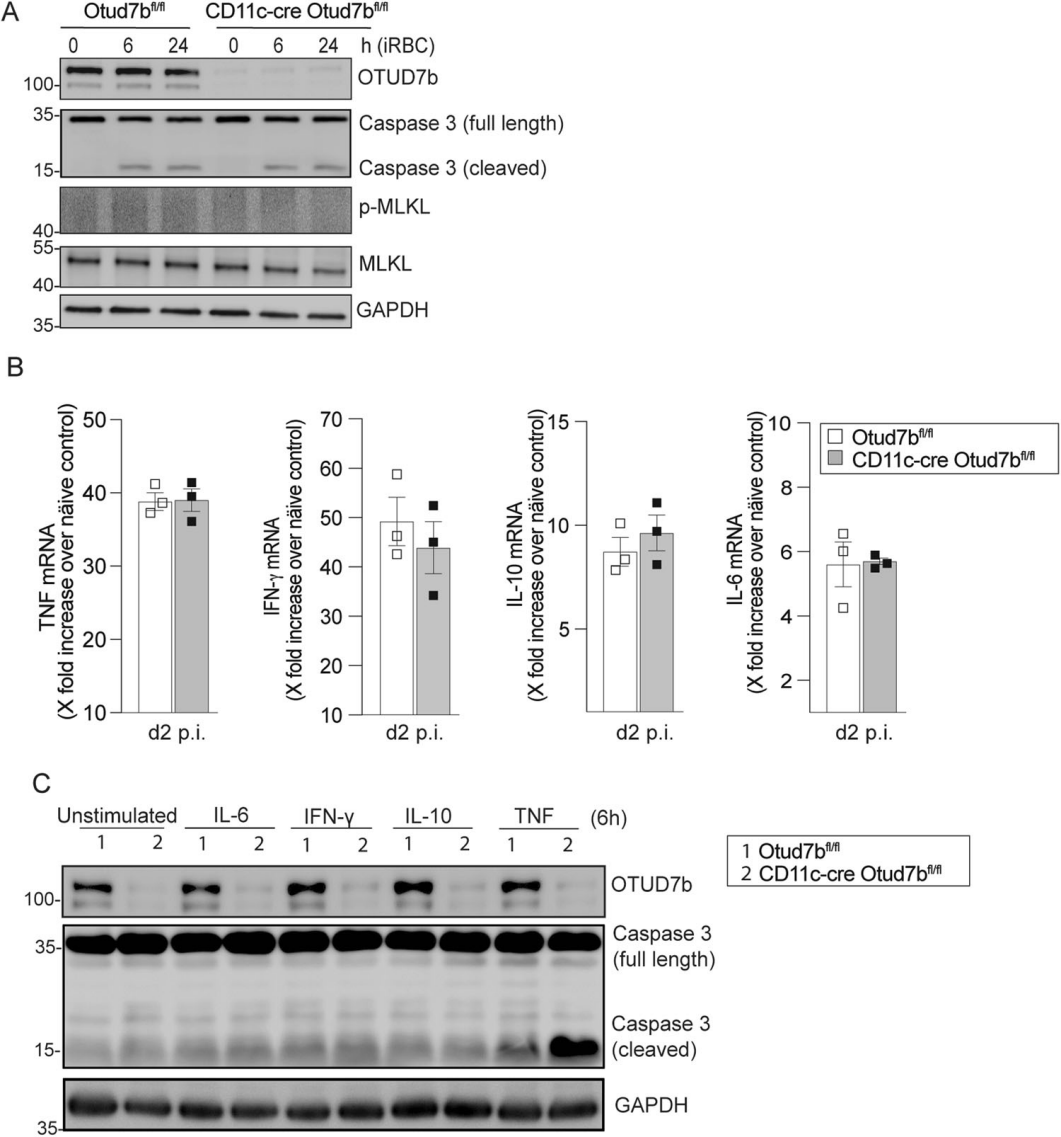
OTUD7b confers protection against apoptosis upon TNF stimulation. **A** Bone marrow-derived dendritic cells (BMDCs) were incubated with *Pb*A-infected RBCs at a ratio of 1:3 (DC:iRBCs). Cells were harvested at indicated timepoints. Isolated proteins were analyzed by WB for the indicated proteins. Representative WB of individual mice are shown (*n* = 3 per group). **B** Otud7b^fl/fl^ and CD11c-Cre Otud7b^fl/fl^ mice were infected i.p. with 1 × 10^6^ iRBCs and spleens were harvested at day 2 p.i. Changes in gene expression of TNF, IL-6, IL-10 and IFN- γ were determined by quantitative real-time PCR (qRT-PCR) and normalized to HPRT. Data represent the fold increase over uninfected controls of same mouse strain (*n* = 3 per group). All bars represent mean values + SEM snd data were statistically analyzed with Student’s t-test. **C** BMDCs were stimulated with 50 ng/ml (500 U/mL) of IL-6, IFN- γ, IL-10 and TNF, respectively, for 6 h. Proteins were analyzed by WB. Representative WB of three biological replicates are shown.

To study the function of OTUD7b in DCs, we established a novel conditional CD11c-Cre Otud7b^fl/fl^ mouse strain (Supplementary Fig. 1A) with specific deletion of OTUD7b in DCs, (Supplementary Fig. 1B, C). CD11c-Cre Otud7b^fl/fl^ mouse developed normally with no alteration in the composition of splenic immune cells including DC subsets (Supplementary Fig. 1D, Fig. 2C). Upon *Pb*A infection, all Otud7b^fl/fl^ mice developed clinical symptoms of ECM at day 7 post infection (p.i.), while all CD11c-Cre Otud7b^fl/fl^ mice remained healthy (Fig. 1A). Evans blue permeability assay showed extensive BBB damage in Otud7b^fl/fl^ mice but not in CD11c-Cre Otud7b^fl/fl^ mice at day 7 p.i. (Fig. 1B). Giemsa-stained thin blood films showed higher parasite loads in CD11c-Cre Otud7b^fl/fl^ mice starting at day 3 p.i, (Fig. 1C). Parasitemia further strongly increased in CD11c-Cre Otud7b^fl/fl^ mice up to day 7 p.i. indicating that, if left untreated, these mice would succumb to anemia due to protracted hyperparasitemia at later stages of the infection. Thus, OTUD7b-deficiency in DCs protects mice from ECM and brain pathology but impairs the pathogen control. Flow cytometric analysis showed that the relative (Supplementary Fig. 2B) and absolute (Fig. 1D) numbers of CD11c^+^ DCs were reduced in spleen of CD11c-Cre Otud7b^fl/fl^ mice at days 2 and 7 p.i. In particular, numbers of conventional cDC-1 (CD11c^+^ CD8α^+^) and cDC-2 (CD11c^+^ CD11b^+^) were reduced, while numbers of PDCA-1^+^ plasmacytoid DCs did not differ between the two mouse strains. (Fig. 1E).

In addition to reduced DC numbers, IL-12 production of CD11c^+^ DCs was reduced in spleens of CD11c-cre Otud7b^fl/fl^ as early as day 2 p.i. (Supplementary Fig. 2H). The expression levels of MHC-I, MHC-II and the T cell co-stimulatory molecules CD80 and CD86 were similar on splenic OTUD7b-competent and -deficient DCs at days 2 and 7 p.i. (Supplementary Fig. 3A) Interestingly, the number of Annexin-V/7AAD^+^ DCs were significantly higher in CD11c-Cre Otud7b^fl/fl^ mice starting day 2 p.i. (Fig. 1F, G).

In concurrence to reduced numbers and IL-12 production of DCs, the relative and absolute numbers of total CD8^+^ T cells (Supplementary Fig. 3B) and GAP50_41–48_ (parasite specific)- CD8^+^ T cells [40] (Supplementary Fig. 3C) were significantly reduced in the spleens of CD11c-Cre Otud7b^fl/fl^ mice at day 7 p.i. Additionally, IFN-γ and Granzyme B production of these parasite-specific splenic CD8^+^ T cells were also significantly reduced (Supplementary Fig. 3D, E). Consequently, intracerebral accumulation of CD8^+^ T cells, the major driver of ECM-associated pathology and death [38, 39], were also reduced in the brains of CD11c-Cre Otud7b^fl/fl^ mice (Supplementary Fig. 4-C). Taken together, DC-specific OTUD7b inhibits death of DCs and fosters IL-12 production of DCs during *Pb*A infection, which leads to effective priming and intracerebral accumulation of pathogen-specific CD8^+^ T cells leading to disruption of the BBB and development of ECM.

### OTUD7b prevents TNF-induced apoptosis of DCs

Since DC apoptosis can be induced either by *Plasmodium*-infected red blood cells (iRBCs) or in response to cytokine [41, 42], we investigated whether the increased cell death of OTUD7b-deficient DCs was mediated by iRBCs (Fig. 2A) or cytokines upregulated during infection (Fig. 2B). Western blot (WB) analysis revealed that levels of cleaved caspase-3 were equally low in iRBC-exposed bone marrow-derived DCs (BMDCs) of both genotypes. However, interestingly, only stimulation with TNF, but not with IL-6, IFN- γ or IL-10 induced apoptosis of OTUD7b-deficient BMDCs as indicated by increased levels of cleaved caspase-3, while the induction of apoptosis in OTUD7b-sufficient BMDCs was comparably weak (Fig. 2C). We note that an equal increase in TNF levels was observed in the serum (Supplementary Fig. 5A) and spleen (*TNF* mRNA) of CD11c-Cre Otud7b^fl/fl^ and Otud7b^fl/fl^ at day 2 post-*Pb*A infection compared to uninfected mice (Fig. 2C). This indicates that DC-specific OTUD7b-deficiency does not regulate overall TNF production, but implies that the increase in DC death of CD11c-Cre Otud7b^fl/fl^ mice is due to the increased sensitivity of OTUD7b-deficient DCs to TNF-induced apoptosis in the presence of similar amounts of TNF.

### OTUD7b stabilizes TRAF2 by cleaving K48-linked polyubiquitin chains and prevents DC apoptosis

**Fig. 3:**
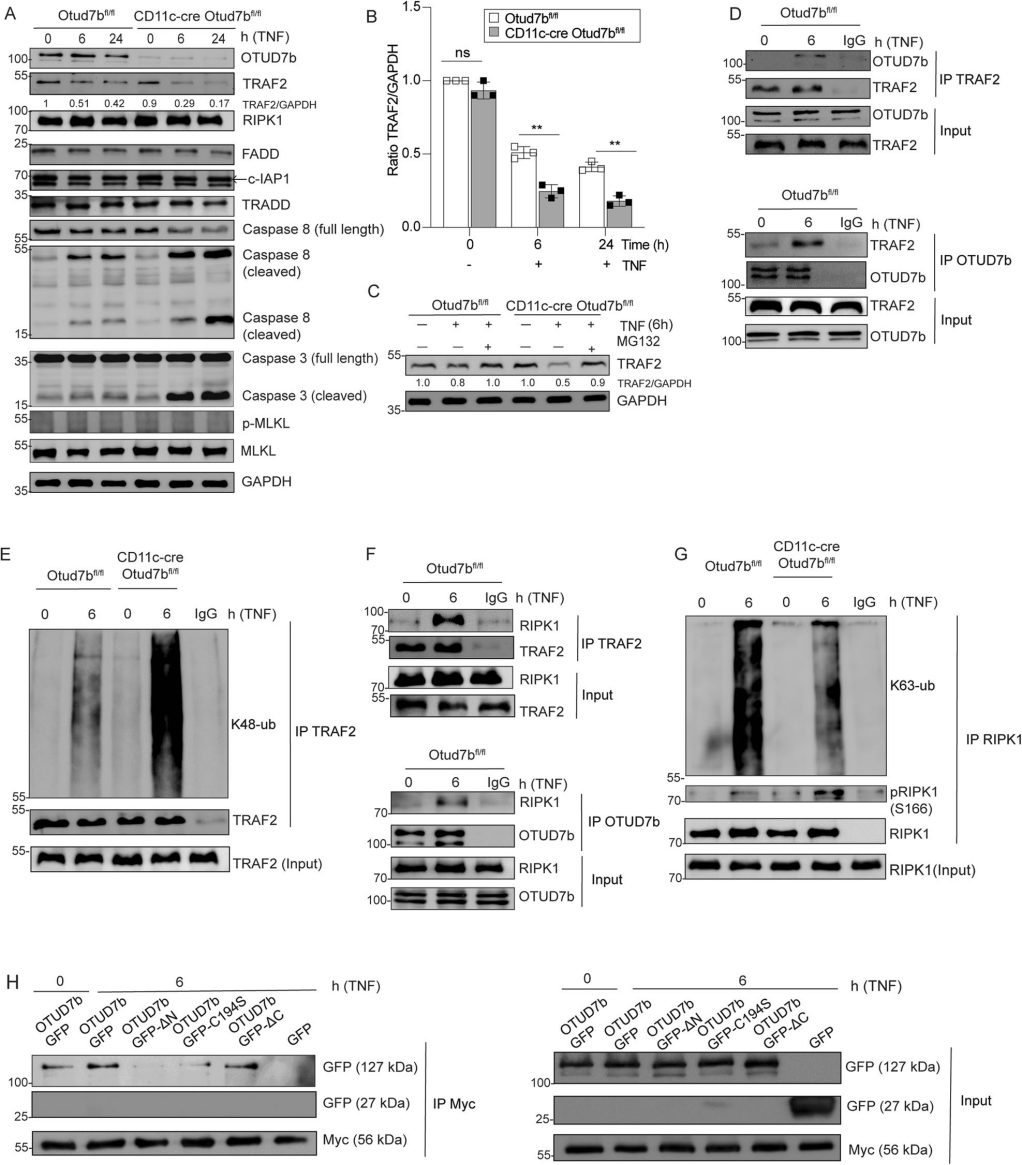
OTUD7b reduces degradation of TRAF2 and diminishes apoptosis. **A**–**C** OTUD7b-sufficient and -deficient BMDC were either stimulated with 50 ng/ml of TNF (**A**, **B**) or a combination of 50 ng/ml of TNF and MG132 (10 µM) (C). **A** Cells were harvested after 0 (unstimulated), 6 and 24 h and stained for the indicated proteins by WB. **B** Relative levels of TRAF2 normalized to GAPDH. All bars represent mean ratio + SEM (*n* = 3, per group and per timepoint), (Student’s t-test, **p < 0.01). **C** WB analysis of TRAF2 protein levels relative to GAPDH upon stimulation with TNF and treatment with MG132 as indicated. The ratio of TRAF2 and GAPDH is shown for all samples. **D**–**G** Protein lysates of unstimulated and TNF-stimulated (50 ng) OTUD7b-sufficient and -deficient BMDCs were immunoprecipitated with either anti-TRAF2 (**D**: upper panel, **E**, **F**: upper panel), anti-OTUD7b (**D** and **F**: lower panel) and anti-RIPK1 (**G**) antibodies, respectively, and immunoblotted for the indicated molecules. Representative blots from one of three experiments are shown. **H** HEK-293T cells were transiently co-transfected with Myc-TRAF2 and GFP-OTUD7b, GFP-OTUD7bΔN (lacking N terminus Ub binding domain), GFP-OTUD7b C194S (catalytically inactive mutant), GFP-OTUD7bΔC (lacking **C** terminus zinc finger region). After TNF stimulation for 6 h, lysates were immunoprecipitated with anti-Myc antibody. Immunoprecipitates were immunoblotted for GFP. Representative blots from one of three experiments are shown.

TNF stimulation led to increased levels of cleaved caspase-3 indicating induction of apoptosis in OTUD7b-deficient BMDCs. However, no phosphorylation of MLKL, i.e. induction of necroptosis, was observed in both genotypes (Fig. 3A). Further WB analysis of TNF stimulated BMDCs showed reduced levels of TRAF2 and increased levels of active caspase 8 in OTUD7b-deficient as compared to OTUD7b-sufficient BMDCs. No difference was observed in the levels of other members of complex-II including c-IAP1, FADD and TRADD (Fig. 3A, B) or cell surface expression of TNFR1 between the two genotypes (Supplementary Fig. 5B).

Inhibition of proteasome in TNF-stimulated BMDCs restored TRAF2 levels of OTUD7b-deficient BMDC demonstrating that OTUD7b prevented proteasomal degradation of TRAF2 (Fig. 3C). Our co-immunoprecipitation experiments showed induced interaction of OTUD7b with TRAF2 upon TNF stimulation (Fig. 3D), Since proteasomal degradation of the substrate is mediated either by K11- or K48-linked polyubiquitin chains, we next determined K48- and K11-linked polyubiquitin chains on TRAF2. WB analysis of the TRAF2 immunoprecipitate showed enhanced K48-linked polyubiquitin chains on TRAF2 in OTUD7b-deficient BMDCs (Fig. 3E), however, the levels of K11 polyubiquitin chains on TRAF2 did not differ between the two groups (Supplementary Fig. 5C), indicating that OTUD7b specifically cleaves K48-linked polyubiquitin chains from TRAF2.

TNF stimulation also resulted in an interaction between TRAF2-RIPK1 and RIPK1-OTUD7b (Fig. 3F). K63-linked polyubiquitination of RIPK1 at lysine 376 [43, 44] prevents autophosphorylation of RIPK1 at serine166, inhibits RIPK1 kinase activity and thereby inhibits induction of cell death [45]. In good agreement, reduced K63-polyubiquitination of RIPK1 and increased phosphorylation of RIPK1 at serine166 was observed in OTUD7b-deficient as compared to OTUD7b-sufficient BMDCs (Fig. 3G). These data indicate that the reduction of TRAF2 K48-polyubiquitination and proteasomal degradation by OTUD7b prevents the pro-apoptotic function of RIPK1 by sustaining RIPK1 K63-polyubiqutination and reducing its serine166 phosphorylation.

It is noteworthy that upon stimulation with FasL and TRAIL, respectively, enhanced TRAF2 degradation and increased levels of cleaved caspase-3 were detected in OTUD7b-deficient BMDCs suggesting that in addition to TNF, OTUD7b inhibits TRAF2-mediated Fas- and TRAIL-induced apoptosis (Supplementary Fig. 5D, E).

Immunoprecipitation experiments showed that the interaction between TRAF2 and OTUD7b was completely abolished by deletion of the N-terminal UBA domain. Interestingly, the catalytic inactive OTUD7b mutant also showed reduced binding to TRAF2, while no difference was observed with the C-terminal zinc finger mutant. Thus, both the UBA domain and the catalytic C194 site of OTUD7b are required for interaction with TRAF2 (Fig. 3H).

### DC-specific OTUD7b augments NF-κB and MAP kinase signaling by stabilizing TRAF2

**Fig. 4:**
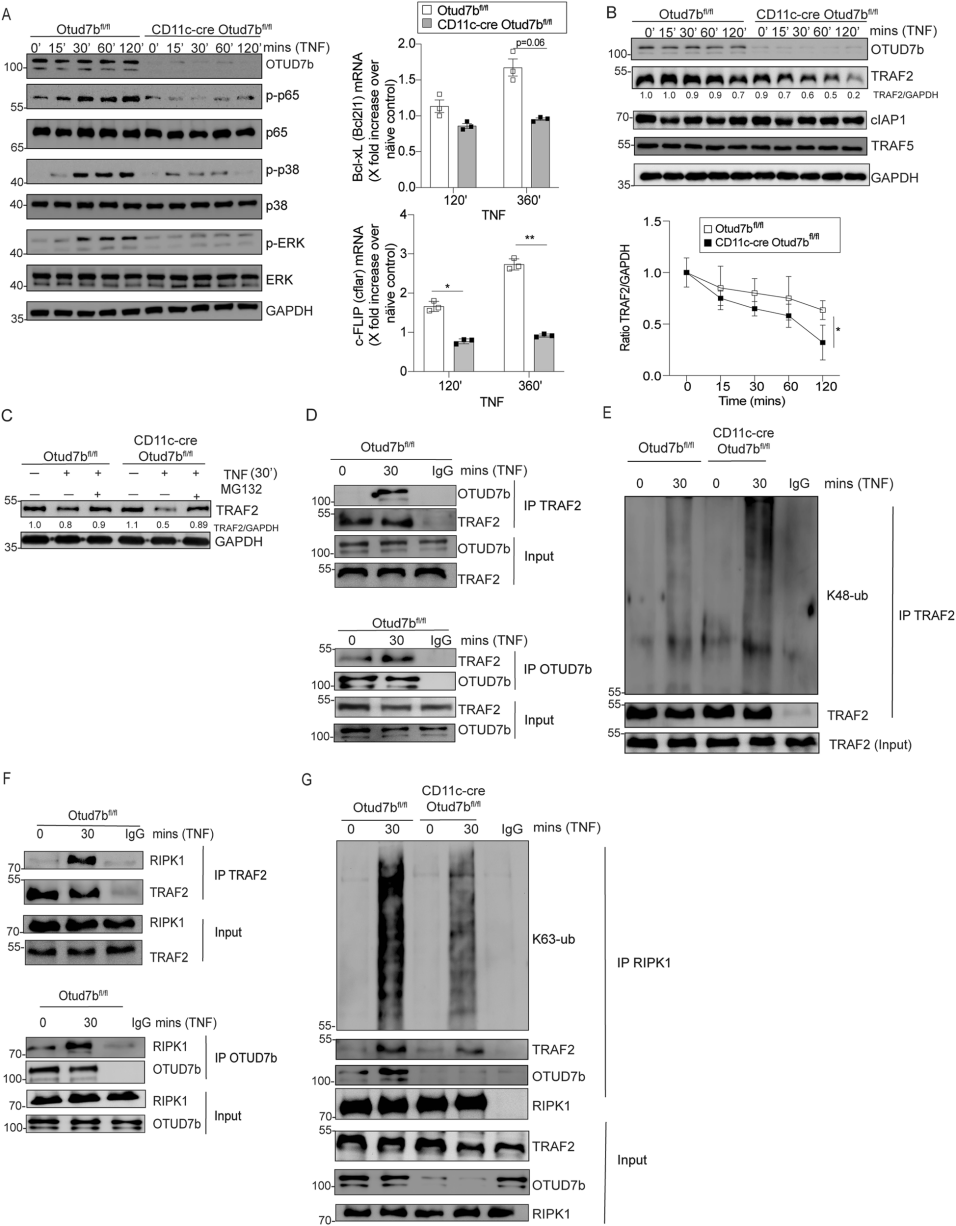
OTUD7b inhibits TRAF2 degradation, increased activation of NF-κB and MAP kinase signaling and expression of anti-apoptotic genes early after TNF stimulation. Increased TRAF2 degradation impairs activation of NF-κB and MAP kinase signaling. **A**, **B** OTUD7b-sufficient and -deficient BMDC were stimulated with 50 ng/ml of TNF. Cells were harvested after 0 (unstimulated), 15, 30, 60 and 120 mins, respectively, and stained for the indicated proteins by WB (**A**, left panel; **B**, upper panel). RT-qPCR analysis of relative gene expression of *Bcl2l1* and *cflar* at 120 and 360 min after TNF stimulation (**A**, top right panel). Mean ± SEM is shown (*n* = 3 per group and timepoint). In B (lower right panel), relative protein levels of TRAF2 determined by WB analysis and normalized to GAPDH are shown. In **A** and **B**, all bars represent mean ratio ± SEM (*n* = 3 per group and timepoint), (Student’s t-test, *p < 0.05). **C** OTUD7b-sufficient and -deficient BMDC were either stimulated with 50 ng/ml of TNF or a combination of TNF and MG132 (10 µM) as indicated. Cells were harvested from unstimulated samples and after 30 min of TNF stimulation. Isolated proteins were analyzed as indicated by WB. **D**–**G** Protein lysates of unstimulated and TNF stimulated (50 ng) OTUD7b-sufficient and -deficient BMDC were immunoprecipitated with anti-TRAF2 (**D**: upper panel, **E**, **F:** upper panel), anti-OTUD7b (**D**, **F**: lower panel) and anti-RIPK1 antibodies (**G**), respectively, and immunoblotted for the indicated proteins. In (**A**–**G**), representative WBs from one of three experiments each are shown.

In addition to cell death, TNF can cause inflammation by activating the NF-κB and MAP-kinase signaling pathways. OTUD7b-deficiency impaired activation of NF-κB and MAPK pathways in comparison to OTUD7b-sufficient BMDCs upon TNF stimulation as shown by reduced levels of p-p65, p-p38 and p-ERK (Fig. 4A, left panel). Transcription factor NF-κB regulates the expression of anti-apoptotic genes [21]. qRT-PCR analysis showed reduced expression of anti-apoptotic Bcl-xL (Bcl2l1) and cFLIP (cflar) in OTUD7b-deficient BMDCs (Fig. 4A, right panel). In line with our data after long-term exposure to TNF (Fig. 3A–G), we observed (i) enhanced proteasomal degradation of TRAF2 (Fig. 4B and C), (ii) TNF-induced OTUD7b-TRAF2 interaction (Fig. 4D) (iii) increased levels of K48-linked polyubiquitin chains on TRAF2 (Fig. 4E), (iv) interaction of TRAF2 with RIPK1 and OTUD7b with RIPK1 (Fig. 4F), and consequently, (v) reduced K63-linked polyubiquitination of RIPK1 (Fig. 4G). Collectively, our data identify OTUD7b as a master regulator of both early pro-inflammatory NF-κB and MAPK signaling and late cell death (apoptosis) responses in DCs upon TNF stimulation.

### Prevention of TNF-induced DC apoptosis renders CD11c-cre Otud7b^fl/fl^ mice fully susceptible to ECM

**Fig. 5:**
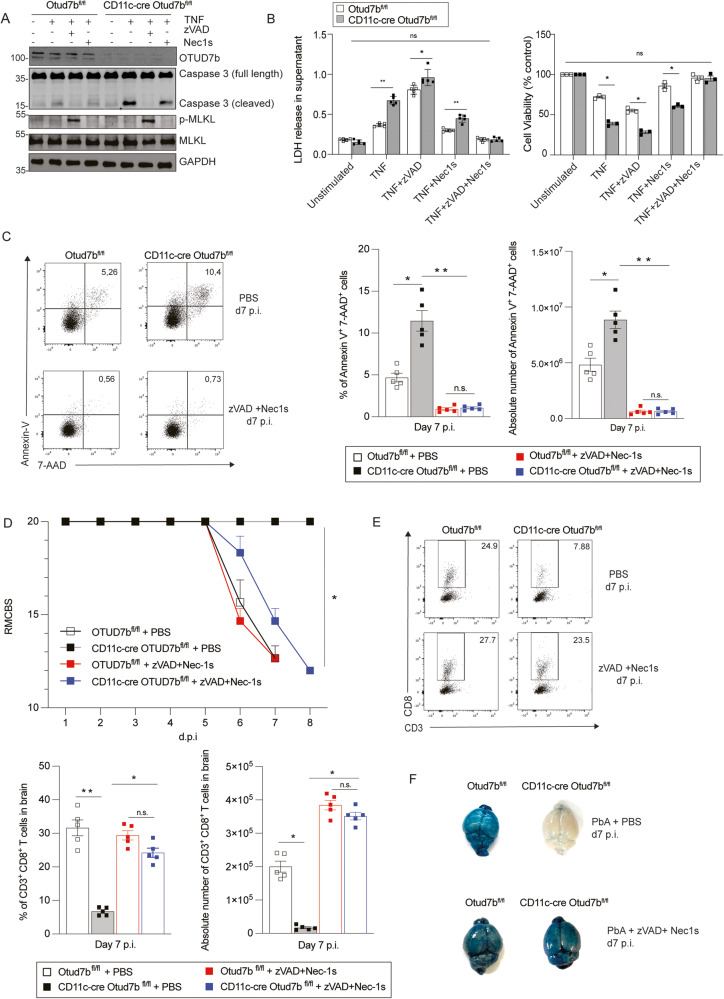
Inhibition of dendritic cell apoptosis in CD11c-Cre Otud7b^fl/fl^ mice fosters CD8^+^ T cells responses and renders mice susceptible to ECM. **A**, **B** OTUD7b-sufficient and -deficient BMDC were pre-treated with zVAD-FMK (50 µM) and Nec-1s (30 µM), 90 minutes prior to TNF stimulation (50 ng). **A** Cells were harvested after 6 h for WB analysis of the indicated proteins. **B** Left panel: LDH release from cells was quantified in the supernatant of unstimulated and stimulated cells. (*n* = 4 per condition). Right panel: The percentage of living cells was determined by a cell viability MTT assay (*n* = 3 per group and condition). For LDH release assay values were expressed as absorbance at 490 nm and for MTT assay values were calculated as percentage viability compared to untreated controls. All bars represent mean values ± SEM (Student’s t-test, *p < 0.05, **p < 0.01). **C**–**F** Otud7b^fl/fl^ and CD11c-Cre Otud7b^fl/fl^ mice were injected i.v. with a combination of zVAD-FMK and Nec-1s (6 mg/kg and 4 mg/kg respectively) or PBS + DMSO, 90 minutes before *Pb*A infection. Inhibitor treatment was continued daily up to day 7 p.i. **C** Representative dot plots (left panel), relative cell numbers (center panel) and absolute cell number (right panel) of Annexin V/7AAD-positive CD11c^+^ cells in spleen (*n* = 5 per group) are shown. All bars represent mean values ± SEM (Student’s t-test, *p < 0.05, **p < 0.01). **D** RMCBS scores of the indicated groups of *Pb*A -infected mice were determined daily (*n* = 5 per group), (Mann–Whitney U test, *p < 0.05 for all groups vs. PBS-treated CD11c- Cre Otud7b^fl/fl^ mice). **E** Relative and absolute cell numbers of CD8^+^ T cells in the brains at day 7 p.i. (*n* = 5 per group) All bars represent mean values ± SEM (Student’s t-test, *p < 0.05, **p < 0.01). (F) Representative images of Evans blue assay. For each group one of 4 mice analyzed is shown. **C**–**E** All bars represent mean values + SEM (Student’s t-test, *p < 0.05, **p < 0.01). All WBs are representative of one of three independent experiments. TNF tumor necrosis factor, Nec-1s Necrostatin 1 s, LDH lactate dehydrogenase, BMDC Bone marrow derived dendritic cell.

To functionally validate that the enhanced cell death of OTUD7b-deficient BMDCs was caused by apoptosis, TNF-stimulated BMDCs of Otud7b^fl/fl^ and CD11c-Cre Otud7b^fl/fl^ mice were treated with apoptosis (pan caspase inhibitor-zVAD-FMK) and apoptosis/necroptosis (RIPK1 inhibitor Nec-1s) inhibitors, respectively. zVAD-FMK treatment prevented apoptosis of both OTUD7b-sufficient and -deficient BMDCs, as indicated by the absence of cleaved caspase-3 (Fig. 5A). However, zVAD-FMK treatment induced de novo activation of necroptosis with induction of p-MLKL (Fig. 5A), increase of LDH release (Fig. 5B, left panel) and reduced cell viability (Fig. 5B, right panel). Treatment with the RIPK1-inhibitor Nec-1s slightly reduced caspase-3 cleavage and LDH release and increased cell viability in both OTUD7b-sufficient and -deficient BMDCs (Fig. 5A, B) without abolishing the difference between the two groups (Fig. 5B). Treatment of TNF-stimulated BMDCs with a combination of zVAD-FMK and Nec-1s completely prevented cell death in both genotypes (Fig. 5A, B). Collectively, OTUD7b inhibits both RIPK1-dependent and -independent apoptosis in DCs

To validate these findings in vivo, *Pb*A-infected Otud7b^fl/fl^ and CD11c-Cre Otud7b^fl/fl^ mice were treated with a combination of zVAD-FMK and Nec-1s. Inhibitor treatment reduced DC death to equally low levels in both genotypes (Fig. 5C) and rendered CD11c-Cre Otud7b^fl/fl^ fully susceptible to ECM (Fig. 5D). Furthermore, zVAD-FMK/Nec-1s treatment of CD11c-Cre Otud7b^fl/fl^ mice increased numbers of intracerebral CD8^+^ T cells to the same level as in OTUD7b-competent control mice (Fig. 5E) and resulted in the loss of BBB integrity (Fig. 5F).

### Ablation of TRAF2 induces DC apoptosis and protects mice from ECM

**Fig. 6:**
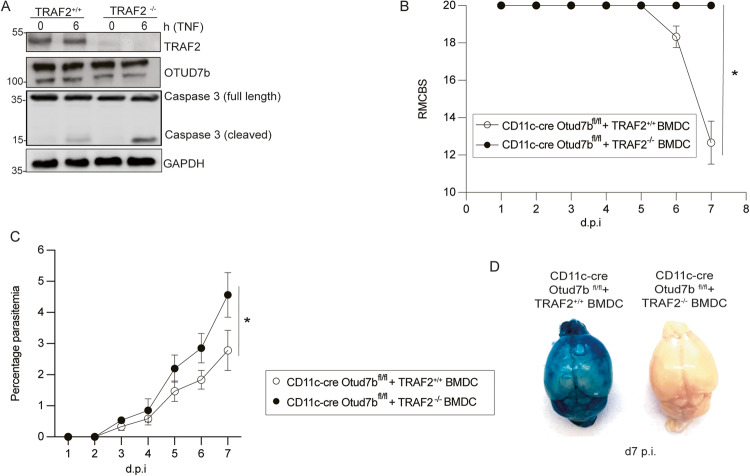
TRAF2 deletion enhances apoptosis of TNF-stimulated OTUD7b-competent BMDC and protects mice from ECM. *Traf2* was deleted in BMDC using CRISPR/Cas9. **A** TRAF2-sufficient and -deficient BMDC were either stimulated with 50 ng/mL of TNF for 6 h or left untreated (0 h). Cells were harvested at the respective time points and stained for the indicated proteins by WB. **B**–**D** TRAF2-sufficient and -deficient BMDC (1×10^6^ cells per recipient) were i.v. adoptively transferred into CD11c-Cre Otud7b^fl/fl^ mice followed by i.p. injection with 1×10^6^ *Pb*A-infected RBCs. **B** Mice were monitored once per day until day 7 p.i. for neurological signs of ECM according to the RMCBS scale (Mann–Whitney U test, *n* = 10 per group, *p < 0.05). **C** Peripheral blood parasitemia was determined daily during the course of infection using Giemsa-stained thin blood smears. Data represent mean values + SEM (*n* = 5 per group, Student’s t-test with *p < 0.05). **D** Representative images of Evans blue assay performed at day 7 p.i. Mice were injected i.v. with Evans blue dye and perfused with 0.9% saline 1 h postinjection. Brains were isolated and photographed. Representative images of one of two independent experiments with 5 mice per group each are shown.

To validate that reduced TRAF2 levels lower the threshold for TNF-mediated cytotoxicity in OTUD7b deficient DCs, we generated TRAF2-deficient BMDCs by CRISPR/Cas9. TRAF2-sufficient and -deficient BMDC expressing OTUD7b were stimulated with TNF. Similar to OTUD7b-deficient DCs, TRAF2-deficient DCs showed increased levels of cleaved caspase-3 after TNF stimulation. (Fig. 6A). To further translate the critical role of TRAF2 for survival of TNF-stimulated BMDCs to in vivo conditions, we adoptively transferred TRAF2-sufficient and -deficient BMDCs to *Pb*A-infected CD11c-Cre Otud7b^fl/fl^ mice. While CD11c-Cre Otud7b^fl/fl^ recipients of TRAF2-deficient BMDC developed no clinical signs of ECM and harbored high parasite load, CD11c-Cre Otud7b^fl/fl^ mice supplemented with TRAF2-sufficient BMDC developed ECM with severe clinical symptoms, disruption of the BBB and a lower parasitemia (Fig. 6B–D). Thus, TRAF2-expressing but not TRAF2-deficient adoptively transferred OTUD7b-competent-DCs rendered CD11c-Cre Otud7b^fl/fl^ mice fully susceptible to ECM further indicating that OTUD7b-dependent TRAF2 stabilization is decisive for the prevention of apoptosis and critical for the induction of ECM.

### OTUD7b prevents DC death in human monocyte-derived DCs

**Fig. 7:**
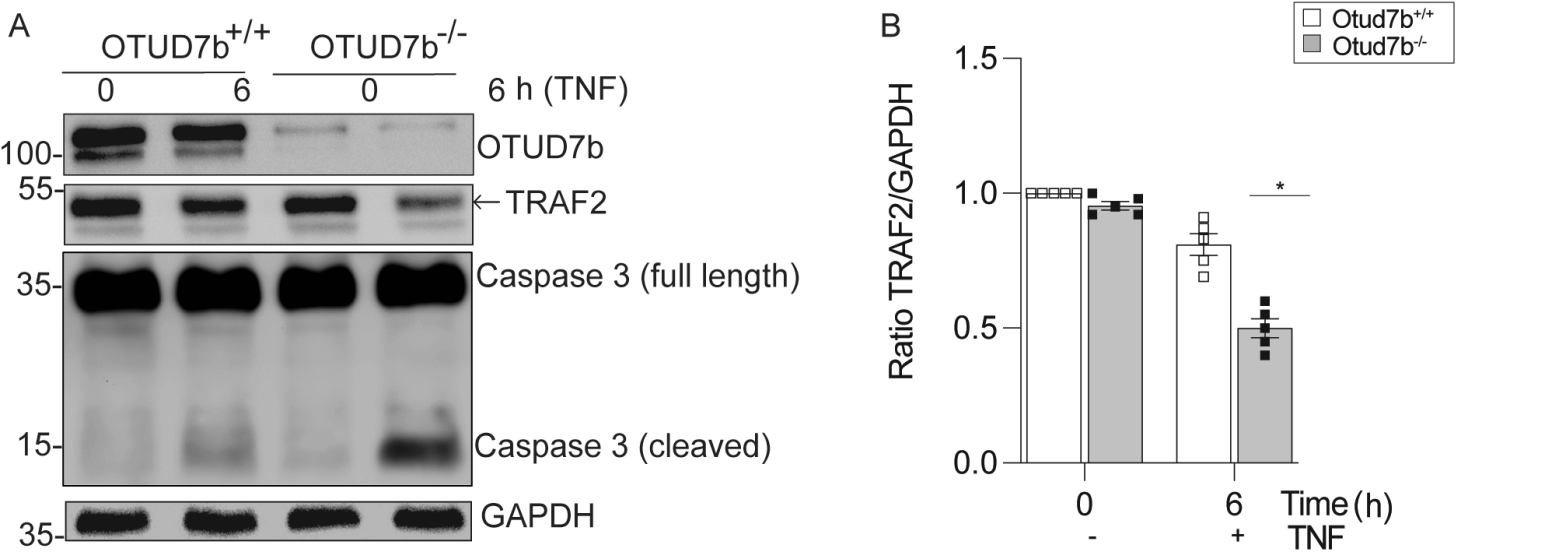
Increased apoptosis of human OTUD7b-deficient monocyte-derived dendritic cells is TRAF2 mediated. *Otud7b* was deleted in human monocyte-derived DCs using CRISPR/Cas9. **A** OTUD7b-sufficient and -deficient human monocyte-derived DCs were stimulated with 50 ng/mL of TNF for 6 h or left unstimulated (0 h). Cells were harvested at the respective time points and stained for the indicated molecules by WB. **B** The ratio of TRAF2 and GAPDH proteins determined by WB are shown. All bars represent mean ± SEM (Student’s t-test, *n* = 5 per group, *p < 0.05). mo-DC: monocyte derived dendritic cell.

To translate our findings to humans, we generated OTUD7b-deficient primary human monocytes by CRISPR/Cas9 and differentiated control and OTUD7b-deficient monocytes to DCs. Similar to murine BMDCs, OTUD7b-deficiency of human DCs enhanced TRAF2 degradation and increased activation of caspase-3 upon stimulation with TNF (Fig. 7A, B). This illustrates a conserved function of OTUD7b for the prevention of TNF-induced apoptosis in human and murine DCs.

## Discussion

**Fig. 8:**
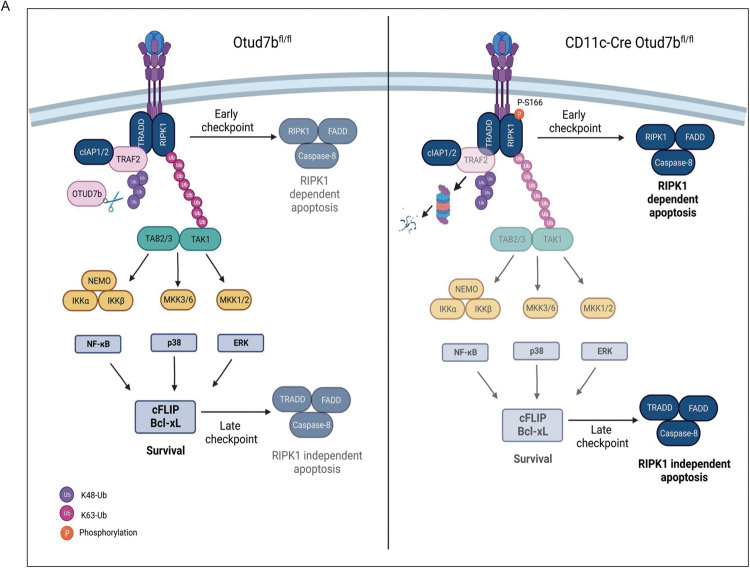
Schematic representation of TNF signaling by OTUD7b in DCs. OTUD7b cleaves K48-linked polyubiquitin chains from TRAF2 and prevents its proteasomal degradation. The stabilized TRAF2 facilitates K63-linked polyubiquitination of RIPK1, which mediates (i) activation of NF-κB and MAP kinases pathway leading to the expression of anti-apoptotic molecules cFLIP and Bcl-xL. cFLIP and Bcl-xL serve as late checkpoint inhibitors of RIPK1-independent apoptosis (ii) K63-linked polyubiquitination of RIPK1 prevents its autophosphorylation at S166 and formation of the death inducing cytosolic complex II comprising of RIPK1, procaspase-8, and FADD which serves as an early checkpoint for the inhibition of RIPK1-mediated apoptosis.

DC intrinsic intertwined regulation of cell death and pro-inflammatory signaling pathways critically determines the outcome of infection. In this regard the cytokine TNF has been identified as a factor promoting the maturation and survival of DCs [17–20]. Here, we identify the DUB OTUD7b as a molecular switch regulating DC fate in response to TNF by fostering TNF-induced pro-inflammatory signaling and prevention of cell death (Fig. 8). Engagement of TNFR1 by TNF induces the activation of NF-κB and MAPK pathways, which results in the production of IL-12 [46]. Interestingly, the activation of both the NF-κB and MAPK pathways was reduced in OTUD7b-deficient DCs upon TNF stimulation leading to impaired IL-12 production. TNF activates these pro-inflammatory signaling pathways in a TRAF2-dependent manner within a few minutes after stimulation. Our data show that this pro-inflammatory function of TNF is dependent on the equally rapid interaction of OTUD7b with TRAF2 and OTUD7b mediated K48-deubiquitination and prevention of TRAF2 proteasomal degradation (Fig. 8). Previous reports have shown that OTUD7b prevents proteasomal degradation of the substrate molecules by cleaving K11-linked polyubiquitin chains [47–49]. However, in our study we observed that OTUD7b specifically cleaves K48, but not K11-linked polyubiquitin chains from TRAF2. This is in concurrence with the studies by Hu et al. ^27^ and Tang et al. ^48^ who also show that OTUD7b regulates protein stability by cleaving K48-polyubiquitin chains from TRAF3 and estrogen receptor-α, respectively.

In the TNFR1 signaling pathway, the E3 ligase TRAF2 in cooperation with cIAP1 performs K63-polyubiquitination of RIPK1. However, the role of ubiquitination and deubiquitination of TRAF2 itself is incompletely characterized. With respect to TRAF2 stability, the E3 ubiquitin ligases, cIAP-1, FBXL2, and siah2 conjugate K48-ubiquitin chains on TRAF2 upon TNFR2, LPS and stress/TNFR1 stimulation respectively [50–52]. Li et al. [53] have proposed that K48 ubiquitination of TRAF2 at multiple lysine residues including K31, K119, K14, K148, K184, K196, K196 and K211 might regulate its stability. The only other DUB known to remove K48-ubiquitin chains from TRAF2 is USP 48 [54]. However, in this study the authors showed that USP48 inhibits only JNK signaling upon TNFR1 stimulation in epithelial cells but does not affect NF-κB, MAPK and cell death signaling [54] indicating mutually nonexclusive cell type- and DUB-specific functions of USP48 and OTUD7b with respect to the regulation of TRAF2.

The formation of the apoptosis-inducing complex-IIa (RIPK1-independent) and complex-IIb (RIPK1-dependent) is induced by insufficient pro-survival signals from complex-I [55]. In good agreement, enhanced TNF-induced degradation of TRAF2 due to the absence of OTUD7b led to reduced K63-linked polyubiquitination of RIPK1, increased serine 166 phosphorylation and impaired activation of NF-κB-dependent expression of anti-apoptotic cFLIP and Bcl-xL resulting in increased caspase-dependent DC apoptosis (Fig. 8). Inhibition of RIPK1 kinase activity by Nec-1s slightly reduced levels of cleaved caspase-3 and partially prevented DC death. However, combined pharmacological inhibition of RIPK1 activity (Nec1s) and apoptosis (zVAD-FMK) completely rescued survival of OTUD7b-deficient BMDC in vitro and in vivo indicating that OTUD7b inhibits RIPK1-independent and to a lesser extent RIPK1-dependent DC apoptosis [43]. Interestingly, OTUD7b also prevented FasL- and TRAIL-mediated apoptosis by stabilizing TRAF2 further demonstrating a central role of OTUD7b in the regulation of TRAF2-mediated cell death receptor signaling. However, we only detected K48 but no K11 deubiquitinating activity of OTUD7b on TRAF2 upon TNF stimulation. Of note, it has been reported that TRAF2-deficiency can be compensated for by TRAF5 leading to normal K63-ubiquitination of RIPK1. However, in TNF-stimulated OTUD7b-deficient BMDCs, TRAF5 was not degraded and could not compensate for the degradation of TRAF2 with respect to RIPK1 K63-polyubiqutination.

The N-terminal UBA domain of OTUD7b is essential for interaction with K11-, K48- and K63-linked ubiquitin chains on target proteins and determines target specificity [56]. Deletion of the UBA domain resulted in complete loss of interaction between OTUD7b and TRAF2. In addition, loss of the active catalytic site C194 showed reduction in binding of OTUD7b to TRAF2 upon TNF stimulation, presumably by disruption of the TRAF binding region spanning the catalytic domain of OTUD7b.

The in vivo function of OTUD7b in infectious diseases has been sparsely studied. With respect to bacterial infections studies employing conventional OTUD7b-deficient mice revealed that OTUD7b inhibits B cell responses by deubiquitinating TRAF3 resulting in impaired control of the intestinal *Citrobacter rodentium* [27]. On the contrary, OTUD7b improves T cell-mediated control of *Listeria monocytogenes* by fostering CD8^+^ T cell receptor signaling and activation due to deubiquitination of Zap70 [57]. With respect to viral infection, in vitro studies revealed that OTUD7b impairs innate anti-viral immunity in macrophages by deubiquitinating SQSTM1/p62 during vesicular stomatitis virus infection [58]. Employing for the first time conditional OTUD7b-deficient mice, we identified that in a model of parasite infection DC-specific OTUD7b critically contributes to cerebral inflammation and brain pathology but is required for parasite control.

In ECM, DCs play a central role in the orchestration of the anti-parasitic immune response. Conventional DCs are essential for the priming of pathogenic CD8^+^ T cells [39, 40, 59], which migrate to the brain and induce the disruption of the BBB causing the disease pathology. On the other hand, DCs also prime protective immune cells including CD4^+^ T cells which recognize and destroy infected RBCs [60]. DCs also play a pivotal role in B cell activation, via the production of B cell activating factor (BAFF) [61]. In addition to T and B cells, DCs further interact with other innate immune cells such as natural killer (NK) [62] and γδ T cells [63] during *Plasmodium* infection all of which aid in the control of the parasite.

Upon *Pb*A infection, CD11c-cre Otud7b^fl/fl^ mice harboured reduced number of CD8α^+^ DCs in the spleen compared to Otud7b^fl/fl^ mice. The reduced numbers of CD8α^+^ DCs in CD11c-cre Otud7b^fl/fl^ along with diminished IL-12 production resulted in reduced numbers of IFN-γ− and Granzyme B-producing pathogen-specific CD8^+^ T cells in spleen and brain leading to protection from ECM.

Adoptive transfer of OTUD7b-competent TRAF2-expressing DCs in CD11-Cre Otud7b^fl/fl^ mice, rendered mice fully susceptible to ECM, whereas OTUD7b-competent TRAF2-deficient DCs failed to induce ECM, indicating the importance of the DC-specific OTUD7b/TRAF2 interaction for ECM development of ECM.

Noteworthy, our study shows similar function of OTUD7b for the stabilization of TRAF2 upon exposure to TNF in human DCs as in their murine counterparts.

The major neurological complication of CM is disruption of the BBB and patients surviving CM suffer from long term cognitive deficits. Therefore, clinical validation of drugs targeting OTUD7b such as the recently identified OTUD7B inhibitor 7Bi [64], may aid in preventing BBB dysfunction by dampening DC-dependent T cell responses and could be used as an adjunct therapy in combination with anti-malarial drugs directly targeting the parasite.

### Limitations of the study

This study focusses on the function of OTUD7b in TNF-signaling of DCs, a key pathway in an immunological important cell population. Depending on the inflammatory milieu, which might predominantly activate other signaling pathways, and the respective cell type, which may be more resistant to TNF-induced cell death, the function of OTUD7b may vary.

## Resource availability

### Method details

#### Data Reporting

No statistical methods were used to calculate the sample size

#### Animals

We generated C57BL/6 Otud7b^fl^/^fl^ mouse strain in which exon 7 of *Otud7b* was flanked by LoxP sites. Otud7b^fl^/^fl^ mice were crossed to CD11c-Cre mice to generate CD11c-cre Otud7b^fl^/^fl^ mice. Otud7b^fl^/^fl^ mice were used as controls in all experiments. All animals were maintained and bred under pathogen free conditions. Age and sex matched 8-10 weeks old animals were used for all the experiments.

#### Ethics Statement

All animal experiments were performed in accordance with German Animal Welfare Act in a protocol approved by local authorities (license number- 33.12-42502-04-20/3500).

#### Parasite infection and clinical assessment of ECM

*Plasmodium berghei* ANKA (*Pb*A) was used for mouse infection experiments. Parasite stocks were prepared by injecting C57BL/6 mice with iRBCs. At day 7 p.i., blood was harvested in Alsever’s solution (Sigma-Aldrich) with 10% glycerol. For blood stage infection, mice were infected intraperitoneally (i.p.) with 1×10^6^ iRBCs per mouse.

For clinical evaluation of neurological signs and ECM development, *Pb*A-infected mice were monitored daily and assessed according to the 10 parameters defined in the Rapid Murine Coma and Behavioral Scale (RMCBS) [65]. Mice with a score of less than 10 were sacrificed.

#### In vitro BMDC treatment

At day 9 of culture BMDCS were seeded in 6-well plates at a concentration of 1×10^6^ cells per well with 35 ng/ml of GM-CSF and 100 ng/ml of LPS (Sigma-Aldrich) for 24 h followed by serum starvation. BMDCs were stimulated with 50 ng/ml (500 U/ml) of IL-6, IFN- γ, IL-10, TNF, TRAIL (peprotech) or CD95L (BD Bioscience) for indicated timepoints. Thereafter, cells were harvested for Western blot (WB) analysis. For the analysis of K48- and K11-linked polyubiquitin chains, cells were treated with TNF in the presence of 10 µM MG132. BMDCs were treated with 50 µM of zVAD-FMK and/or with 30 µM of Nec1s beginning 90 minutes before TNF stimulation. The inhibitors were dissolved in DMSO according to manufacturer’s recommendation.

#### Cell culture and transfection

HEK-293T cells (ATCC) were cultured in DMEM medium supplement with 10% FCS and 1% penicillin/streptomycin. STR profiling was used by ATCC for cell line authentication and cultured cells were routinely tested for mycoplasma contamination. The deletion constructs pCMV6-GFP-OTUD7b-ΔN, pCMV6-GFP-OTUD7b-C194S and pCMV6-GFP-OTUD7b-ΔC were generated from pCMV6-GFP-OTUD7b plasmid (Origene) using Q5 site directed mutagenesis kit (NEB). Plasmid constructs were verified by sequencing (Eurofins). The constructs were transiently transfected into HEK-293T cells using Lipofectamine 3000 (Thermo Fisher Scientific) according to manufacturer’s protocol.

#### In vivo Z-VAD-FMK and Nec-1s treatment

Mice were i.v. injected with a combination of zVAD-FMK (6 mg/kg) and Nec-1s (4 mg/kg) diluted in PBS 90 min prior to *Pb*A infection and then once per day for the next 7 days. Control mice were injected with vehicle (DMSO + PBS) followed by *Pb*A infection.

#### Human monocyte derived-DCs

Human blood was obtained from the Blood Transfusion Center of Hannover Medical School and PBMCs were isolated using Ficoll density gradient centrifugation followed by magnetic cell separation of CD14^+^ monocytes (MojoSort, Biolegend). Stable knockdown of OTUD7b was generated using CRISPR/Cas9 system and P3 primary cell 4D-nucleofector X kit (Lonza) according to the manufacturer´s protocol. CD14^+^ monocytes were cultured in DMEM medium supplemented with 50 ng GM-CSF and 50 ng IL-4 for 5 days. Cells were harvested and stimulated as per experimental requirement.

#### Statistics

The statistical significance was determined using software Prism 9 using respective tests as mentioned in the figure legends. P values of ≤0.05 were considered significant. All experiments were performed at least twice.

## Supplementary information

Supplementary materials and methods

Supplementary figures and legends

Original Data File

Checklist

## Data Availability

Any additional information is available from the lead contact upon request.
