# Supplementary material for: The deubiquitinating enzyme OTUD7b protects dendritic cells from TNF-induced apoptosis by stabilizing the E3 ligase TRAF2
Source: Cell Death Dis. 2023 Jul 29;14(7):480. doi: 10.1038/s41419-023-06014-5 (PMC10387084; doi:10.1038/s41419-023-06014-5)
Supplement: Supplementary file 1 — Supplementary materials and methods [file 41419_2023_6014_MOESM1_ESM.docx]

Supplemental Methods

Assessment of parasitemia

Parasitemia was assessed by microscopic examination of Giemsa-stained thin blood smears Blood smears were fixed in absolute methanol and stained with 10% Giemsa solution (Carl Roth) in PBS (Thermo Fisher Scientific). Parasite load was quantified by determining the percentage of iRBCs in a randomized blinded manner under 10 microscopic light fields.

Evans Blue staining

Mice were intravenously (i.v.) injected with 100 μl of 1% solution of Evans Blue dye (Sigma Aldrich) in PBS at day 7 p.i. After 1h, mice were anesthetized with isoflurane and perfused intracardially with 0.9% NaCl. Perfused brains were collected and photographed.

Leukocyte isolation and flow cytometry-

For measurement of splenic leukocytes population, spleens were harvested and homogenized through 50 μm cell strainer. Erythrocytes were lysed with ammonium chloride RBC lysis buffer. Splenocytes were stained with mouse specific fluorochrome-labelled antibodies as described in the supplementary table 1.

For intracerebral leukocytes isolation, anaesthesized mice were perfused intracardially with 0.9% NaCl, brains were isolated and homogenized through 70 μm mesh strainer. Leukocytes were separated using a Percoll^®^ density gradient centrifuge (GE Healthcare) and stained with specific antibodies described in the supplementary table 1. For staining of *Pb*A-specific CD8^+^ T cells PE-conjugated H2-D^b^ SQLLNAKYL pentamer (ProImmune) staining was performed according to manufacturer’s instructions in conjunction with CD8^+^ staining.

For intracellular cytokine staining, splenocytes were stimulated with GAP50 (SQLLNAKYL) peptide (10 µg/ml) or Phorbol 12-myristate 13-acetate (PMA, 20ng/ml) plus ionomycin (1µg/ml) for cytokine production by DCs in RPMI 1640 medium (Thermo Fischer Scientific) at 37°C in the presence of 1 μg/mL Brefeldin A solution (Biolegend) for 4h followed by fixation and permeabilization using cytofix/cytoperm (BD Biosciences). Staining was performed with respective antibodies as described in the supplementary table 1.

Flow cytometric analysis of cell death was performed using a FITC-Annexin V Apoptosis Detection Kit (BioLegend) according to manufacturer’s protocol.

Flow cytometry was performed on a Cytek NL-3000 instrument (Cytek Bioscience) and data were analyzed with FlowJo® software (FlowJo LLC).

Bone marrow-derived dendritic cell (BMDC) culture-

Bone marrow cells were isolated from femur and tibia and differentiated into BMDCs as previously described ^32^ using 35ng/mL of Granulocyte macrophage colony stimulating factor (GM-CSF) (Peprotech), or 200ng/mL of FMS-like tyrosine kinase 3 ligand (Flt3L) (Peprotech). Macrophage colony-stimulating factor (M-CSF) (Peprotech) was used for Bone marrow derived macrophage (BMDM) differentiation. Medium was changed every 3 days and at day 9 cells were harvested and used for experiments. BMDCs and BMDM cultures contained >90 % CD11c^+^ DCs and CD11b^+^ macrophages, respectively, as determined by flow cytometry.

Co-incubation of BMDCs with iRBCs

1x10^6^ BMDCs were seeded per well in a 6-well plate and incubated with intact iRBCs at a ratio of 1:3 (DC:iRBC) for 6 h and 24 h, respectively. Uninfected RBCs from healthy mice were used as controls. Cells were lysed in RIPA lysis buffer after 6 h and 24 h, respectively, followed by WB analysis.

Western Blot analysis

Sample preparation and western blot analysis was performed as described previously ^32^. In brief, equal amounts of protein were separated using SDS-polyacrylamide gel electrophoresis and transferred to polyvinylidene fluoride (PVDF) membranes. After 1 h of blocking, membranes were incubated overnight with primary antibodies described in the supplementary table 2 at 4°C. Blots were developed using Pierce ECL plus kit (Thermo Fisher Scientific) and images were acquired with an Intas chemo cam luminescent image analysis system (INTAS Science Imaging Instruments, Göttingen, Germany). For quantification and analysis, LabImage 1D software was used (Intas Science Imaging Instruments).

Immunoprecipitation

Protein lysates were precleared by incubating with Sepharose G beads (GE healthcare) at 4°C with continuous shaking for 1 h. Lysates were then incubated with specific antibodies for 4°C overnight with shaking. The following day, immune complexes were captured by incubating lysates with Sepharose G beads for 2 h and then washed 3 times with ice cold PBS. Beads were resuspended in 2x lane marker reducing sample buffer and boiled at 99°C for 10 min. After centrifugation, supernatant was collected and used for WB analysis.

Quantitative reverse transcription-PCR (RT-qPCR)

Isolation of mRNA from spleen of uninfected and infected mice was performed using RNAeasy kit (Qiagen) and mRNA was transcribed into cDNA using SuperScript Reverse Transcriptase Kit (Thermo Fisher Scientific). Quantitative RT-PCR was performed with a Lightcycler 480 system (Roche Diagnostics) and TaqMan probes (Thermo Fisher Scientific) described in supplementary table 3. Gene expression were normalized to HPRT and analyzed according to the ΔΔCT threshold cycle method. All data are expressed as fold-increase in mRNA concentration of infected mice over respective uninfected control mice.

Cytometric Bead Array (CBA)

Blood was collected from *Pb*A infected Otud7b^fl/fl^ and CD11c-Cre Otud7b^fl/fl^ mice at day 2 p.i. Serum was separated by centrifugation and CBA analysis was performed using manufacturer’s instruction (BD™ Cytometric Bead Array (CBA) Mouse Inflammation Kit)

MTT assay

BMDCs were plated at a density of 5 x10^4^ cells/well in 96 well plates. Cells were treated with TNF or a combination of TNF+zVAD-FMK, TNF+Nec-1s or TNF+zVAD-FMK+Nec-1s, respectively as mentioned above. After 6h, MTT assay was performed according to manufacturer’s protocol using a Cell Proliferation Kit (MTT) (Merck Millipore).

Lactate dehydrogenase (LDH) assay

LDH enzyme activity was determined in the supernatant from BMDCs stimulated with TNF, TNF+zVAD-FMK, TNF+Nec-1s or TNF+zVAD-FMK+Nec-1s, respectively, for the indicated time points using CyQUANT LDH Cytotoxicity Assay (Thermo Fisher Scientific) according to manufacturer’s protocol.

Magnetic sorting of splenic leukocytes-

Spleens were harvested from Otud7b^fl/fl^ and CD11c-cre Otud7b^fl/fl^ mice and dissociated into single-cell suspension for the isolation of DCs, macrophages, NK cells, T cells, and B cells using cell specific Isolation Kits (STEMCELL Technologies) according to manufacturer’s protocol.

Adoptive transfer of DCs

BMDCs from Otud7b^fl/fl^ mice were isolated and cultured in DMEM medium as mentioned previously. At day 3 of culture, CRISPR/Cas9 mediated knockdown of TRAF2 in BMDC was performed using P4 primary cell 4D-nucleofector X kit (Lonza). For 1x10^6^ cells, 210 pmol of duplexed gRNA was mixed with 70 pmol of Cas9 Nuclease V3 (IDT) for RNP complex synthesis. The complex was mixed with 70 pmol of EE buffer (IDT) to form electroporation mix. Electroporation was performed using preset program DZ100 in Lonza nucleofection system according to the manufacturer’s recommendations. Knockdown of TRAF2 was controled by WB analysis.

For adoptive transfer, TRAF2-competent and TRAF2-deficient BMDC cells were diluted in PBS and 1x10^6^ of the respective BMDCs were injected i.v. into CD11c-cre Otud7b^fl/fl^ mouse.

**Supplementary table 1: Flourochrome conjugated antibodies for flow cytometric analysis**

| Antibody | Manufacturer | Catalogue number |
| --- | --- | --- |
| anti-CD3-FITC | BioLegend | 100204 |
| anti-CD8-BV421 | BioLegend | 100738 |
| anti-CD45-PerCP | BioLegend | 103130 |
| anti-CD11c-PE | eBioscience | 12-0114-83 |
| anti-CD8α-FITC | eBioscience | 11-0081-85 |
| anti-CD11b-PeCy7 | BioLegend | 101216 |
| anti-PDCA-1-APC | BioLegend | 127016 |
| anti-CD86-FITC | BioLegend | 105006 |
| anti-CD80-PE | BioLegend | 104708 |
| anti-MHC-I-PE | BioLegend | 116508 |
| anti-MHC-II-FITC | BioLegend | 107606 |
| anti-NK1.1APCCy7 | BioLegend | 108724 |
| anti-CD11b-APCCy7 | BioLegend | 101226 |
| anti-B220-BV510 | BioLegend | 103247 |
| anti-F4/80-BV421 | BioLegend | 123131 |
| anti-Ly6C-APC | eBioscience | 17-5932-82 |
| anti-mouse IgG-APC | eBioscience | 17-4015-82 |
| anti-Ly6G-PE | eBioscience | 12-9668-82 |
| anti-IL-12-PE | eBioscience | 12-7123-82 |
| anti-IFNγ-PE | eBioscience | 12-7311-82 |
| anti-Granzyme-B-PE | eBioscience | 12-8898-82 |
| anti-Annexin V-APC | BioLegend | 640920 |
| 7AAD-PerCP | eBioscience | 00-6993-50 |

**Supplementary table 2: Primary antibodies used for western blot and immunoprecipitation experiments**

| Antibody | Manufacturer | Catalogue number |
| --- | --- | --- |
| anti-OTUD7b | Proteintech | 16605-1-AP |
| anti-TNFR1 | Proteintech | 21574-1-AP |
| anti-GAPDH | Cell Signaling Technology | 2118 |
| anti-caspase 3 | Cell Signaling Technology | 9662 |
| anti-cleaved caspase 3 | Cell Signaling Technology | 9664 |
| anti-phospho-p65 | Cell Signaling Technology | 3031 |
| anti-IκBα | Cell Signaling Technology | 4812 |
| anti-p65 | Cell Signaling Technology | 8242 |
| anti-phospho-p38 MAPK | Cell Signaling Technology | 9215 |
| anti-p38 MAPK | Cell Signaling Technology | 9212 |
| anti-phospho-p44/42 MAPK | Cell Signaling Technology | 9101 |
| anti-p44/42 MAPK | Cell Signaling Technology | 9102 |
| anti-TRAF2 | Cell Signaling Technology | 4724 |
| anti-RIPK1 | Cell Signaling Technology | 3493 |
| anti-caspase 8 | Cell Signaling Technology | 4790 |
| anti-cleaved caspase 8 | Cell Signaling Technology | 9429 |
| anti-Myc Tag | Cell Signaling Technology | 2276 |
| anti-phospho-RIPK1 Ser166 | Cell Signaling Technology | 31122 |
| anti-TRADD | Cell Signaling Technology | 3694 |
| anti-phospho MLKL S345 | Abcam | ab196436 |
| anti-cIAP1 | Abcam | ab154525 |
| anti-FADD | Abcam | ab124812 |
| anti-ubiquitin Lys48-specific | Merck Millipore, | 05-1307 |
| anti-ubiquitin Lys63-specific | Merck Millipore | 05-1308 |
| anti-ubiquitin Lys11-specific | Merck Millipore | MABS-107-I |
| anti-MLKL | Merck Millipore | MABC604 |
| anti-GFP | Origene | TA150041 |

**Supplementary table 3: TaqMan probes used for qRT-PCR experiments**

| Gene | Manufacturer | Assay ID |
| --- | --- | --- |
| *Bcl2l1* | Thermo Fischer Scientific | Mm00437783 |
| *Cflar* | Thermo Fischer Scientific | Mm01255576 |
| *Hprt* | Thermo Fischer Scientific | Mm01545399 |
| *Ifn-γ* | Thermo Fischer Scientific | Mm00801778 |
| *Il-6* | Thermo Fischer Scientific | Mm00446190 |
| *Tnf* | Thermo Fischer Scientific | Mm00443258 |
| *Il-12* | Thermo Fischer Scientific | Mm99999067 |
| *Il-10* | Thermo Fischer Scientific | Mm00439616 |
