# Supplementary material for: The deubiquitinating enzyme OTUD7b protects dendritic cells from TNF-induced apoptosis by stabilizing the E3 ligase TRAF2
Source: Cell Death Dis. 2023 Jul 29;14(7):480. doi: 10.1038/s41419-023-06014-5 (PMC10387084; doi:10.1038/s41419-023-06014-5)
Supplement: Supplementary file 2 — Supplementary figures and legends [file 41419_2023_6014_MOESM2_ESM.docx]

**Supplementary figures**

**
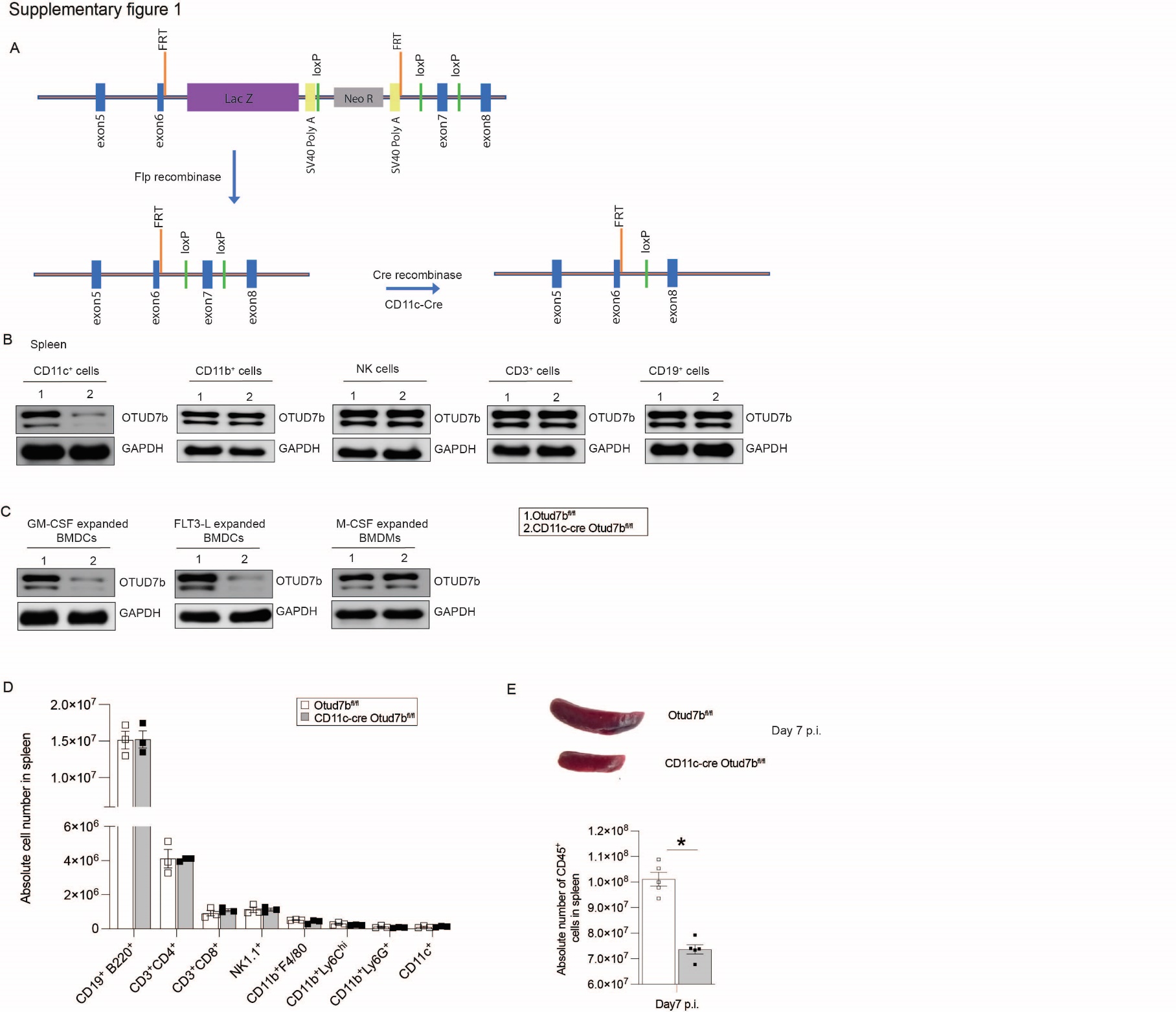
**

Supplementary Figure 1: Characterization of CD11c-Cre Otud7b^fl/fl^ mice

(A) Schematic representation of targeting strategy for the creation of Otud7b ^fl/fl^ mice with LoxP sequences flanking exon 7 of Otud7b gene. Embryonic stem cells derived from C57BL/6 N mice were transfected with the targeting vector and the positive clones were selected for lac Z expression and neomycin resistance. The FRT-flanked NeoR and Lac Z cassettes were subsequently removed by crossing to FLP-deleter strain, to generate Otud7b ^fl/fl^ mice. To generate delete OTUD7b in DCs, Otud7b ^fl/fl^ mice were further crossed to CD11c-Cre mice (49) to generate, CD11c-Cre Otud7b^fl/fl^ mice. (B) CD11c^+^, CD11b^+^, NK1.1^+^, CD3^+^ and CD19^+^ cells were sorted from spleen of uninfected Otud7b^fl/fl^ and CD11c-Cre Otud7b^fl/fl^ mice by magnetic sorting. Expression of OTUD7b was analyzed by WB. (C) Expression of OTUD7b in GM-CSF- and Flt3L-expanded BMDCs and M-CSF-expanded BMDMs from uninfected Otud7b^fl/fl^ and CD11c-Cre Otud7b^fl/fl^ mice was analyzed by WB. (D) Absolute cell numbers of the indicate leukocyte populations in spleen of uninfected Otud7b^fl/fl^ and CD11c-Cre Otud7b^fl/fl^ mice (n=5 per group). (E) Otud7b^fl/fl^ and CD11c-cre Otud7b^fl/fl^ mice were infected i.p. with 1x10^6^ *Pb*A-infected RBCs. Macroscopic image of spleens of Otud7b^fl/fl^ and CD11c-Cre Otud7b^fl/fl^ mice at day 7 p.i. (top). Representative images of one mouse per experimental group (n = 5 per group) are shown. Absolute number of CD45^+^ leukocytes in spleen at day 7 p.i. (bottom). Data represent the mean values + SEM. (Student’s t-test), (n=5 per group, *p<0.05) *Pb*A *Plasmodium berghei* ANKA, p.i. post infection, RBC red blood cell.


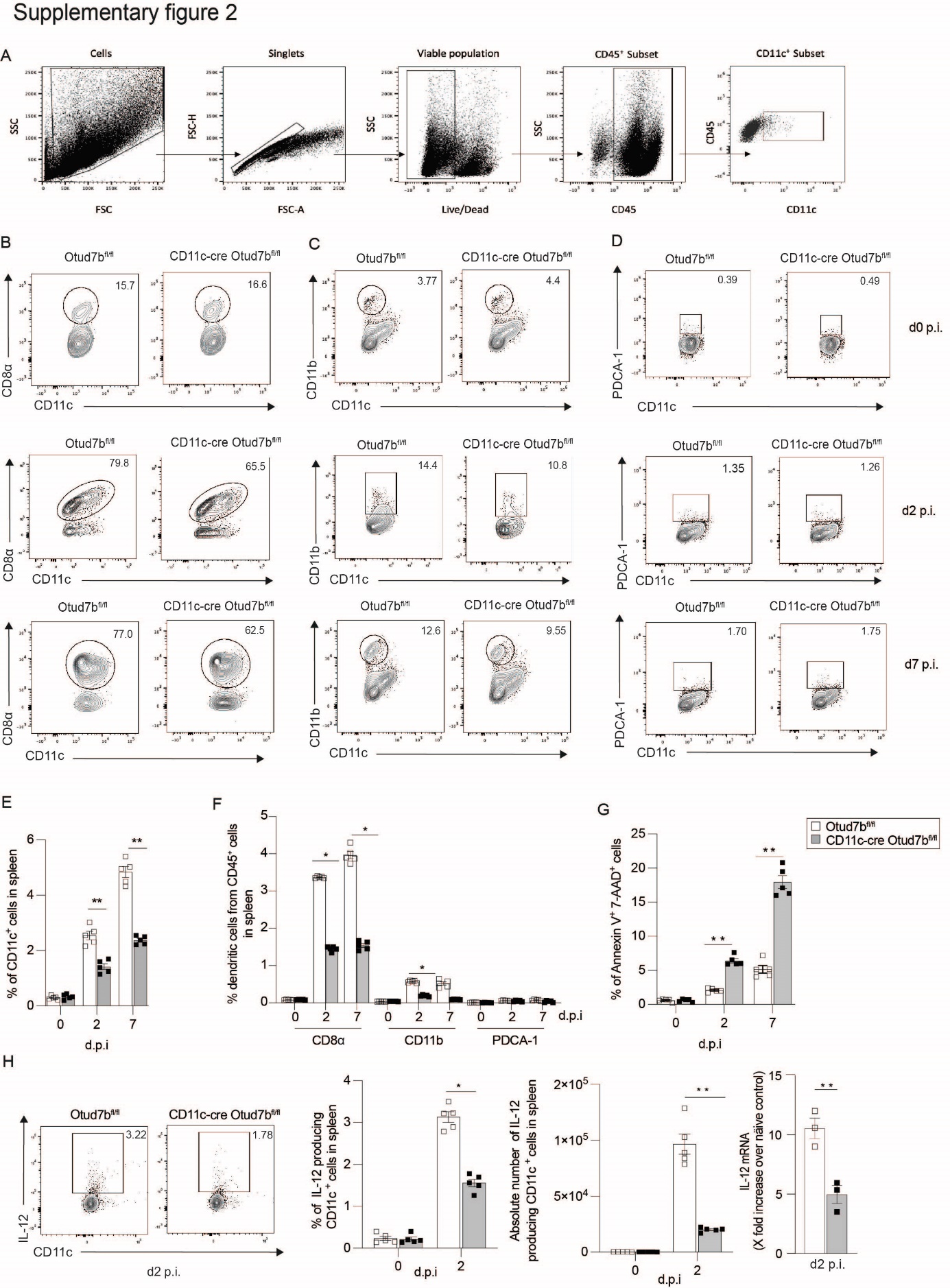


Supplementary Figure 2: Reduced number and impaired IL-12 production of DCs in *Pb*A-infected CD11c-Cre Otud7b^fl/fl^ mice. Alternative: OTUD7b preserves numbers and IL-12 production of DCs upon infection with *Pb*A

Otud7b^fl/fl^ and CD11c-Cre Otud7b^fl/fl^ mice were infected i.p. with 1x10^6^ *PbA*-infected RBCs. Leukocytes were isolated from spleens at days 2 and 7 p.i. and analyzed by flow cytometry (A) Gating strategy for CD45^+^ CD11c^+^ cells. Splenocytes were isolated, red blood cells were lysed and the remaining cells were stained for CD45 and DC-specific markers. Stained cells were analyzed by flow cytometry (B) Representative dot plots of CD11c^+^ CD8α^+^, CD11c^+^ CD11b^+^ and CD11c^+^ PDCA1^+^ DC subtypes at days 0, 2 and 7 p.i. (E) Relative cell numbers of CD11c^+^ DCs in spleen, analyzed at days 0, 2 and 7 p.i. by flow cytometry (n=5 per group). (F) Relative cell numbers of CD11c^+^ CD8α^+^, CD11c^+^ CD11b^+^ and CD11c^+^ PDCA1^+^ DCs at days 0, 2 and 7 p.i. (n=5 per group). (G) Relative number of AnnexinV/7AAD double positive CD11c^+^ DCs in spleens of Otud7b^fl/fl^ and CD11c-Cre Otud7b^fl/fl^ mice in uninfected (d0) and infected (d2 and d7 p.i.) mice (n=5 per group and time point). (H) Representative dot plot (left), relative (middle left panel) and absolute (middle right panel) numbers of PMA plus ionomycin stimulated splenic IL-12-producing CD11c^+^ DCs identified by flow cytometry at days 0 and 2 p.i. (n=5 per group). The right panel shows the fold change of *IL-12* gene expression of infected over uninfected mice of the same mouse strain as determined by qRT-PCR and normalization to HPRT (n=3 per group). (E-H) Data represent the mean values + SEM. (A-C) Student’s t-test, *p<0.05, **p<0.01 *Pb*A *Plasmodium berghei* ANKA, p.i. post infection, RBC red blood cell, qRT-PCR quantitative real-time PCR.


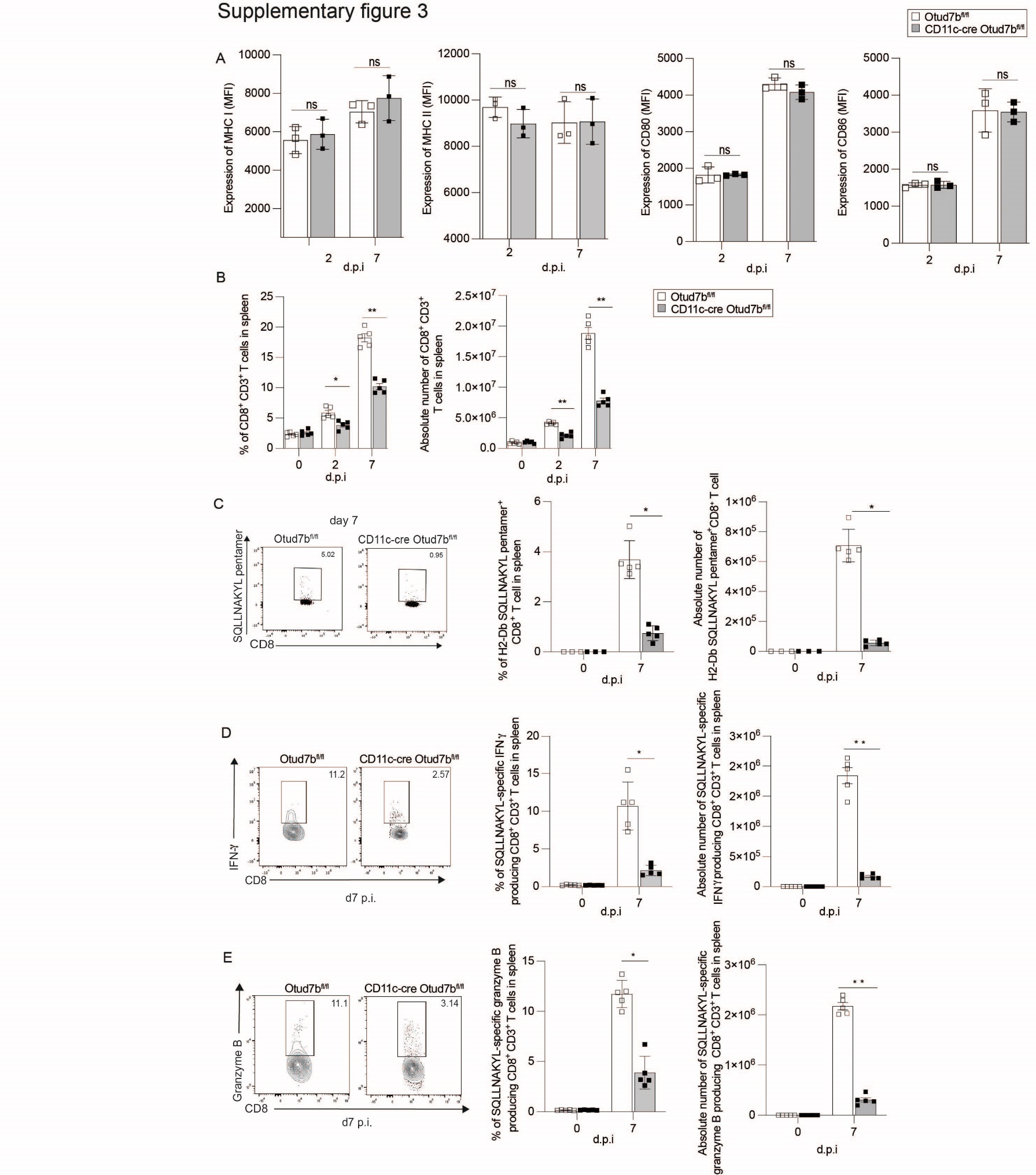
Supplementary Figure 3: Reduced numbers of CD8^+^ T cells in spleens of *Pb*A-infected CD11c-Cre Otud7b^fl/fl^ mice. Alternative: OTUD7b is dispensable for activation of DCs but important for expansion of CD8^+^ T cells in *PbA*-infected mice

Otud7b^fl/fl^ and CD11c-Cre Otud7b^fl/fl^ mice were infected i.p. with 1x10^6^ *Pb*A-infected RBCs. (A) MFI of MHC-I, MHC-II, CD80 and CD86 on CD11c^+^ cells at days 2 and 7p.i. (B) Relative (left panel) and absolute cell number (right panel) of CD3^+^ CD8^+^ T cells in spleens i of uninfected (day 0) and *Pb*A-infected mice at day 7 p.i. (n=5 per group and trime point) (C) SQLLNAKYL pentamer^+^ CD8^+^ T cells population in spleen of *Pb*A-infected mice at day 7 p.i. Representative dot plots (left) relative (center) and absolute (right) cell numbers of H2-D^b^ SQLLNAKYL pentamer^+^ CD8^+^ T cells (n=5, per group) (D, E) Representative dot plots, relative (left) and absolute (right) cell numbers of splenic SQLLNAKYL peptide-stimulated IFN-γ- (D) and Granzyme B- (E) producing CD3^+^ CD8^+^ T cells detected by flow cytometry at days 0 and 7 p.i. (n=3 per group and time point). Data represented as mean values + SEM. (A-E) Student’s t-test, *p<0.05, **p<0.01 *Pb*A *Plasmodium berghei* ANKA, p.i. post infection, RBC red blood cell.


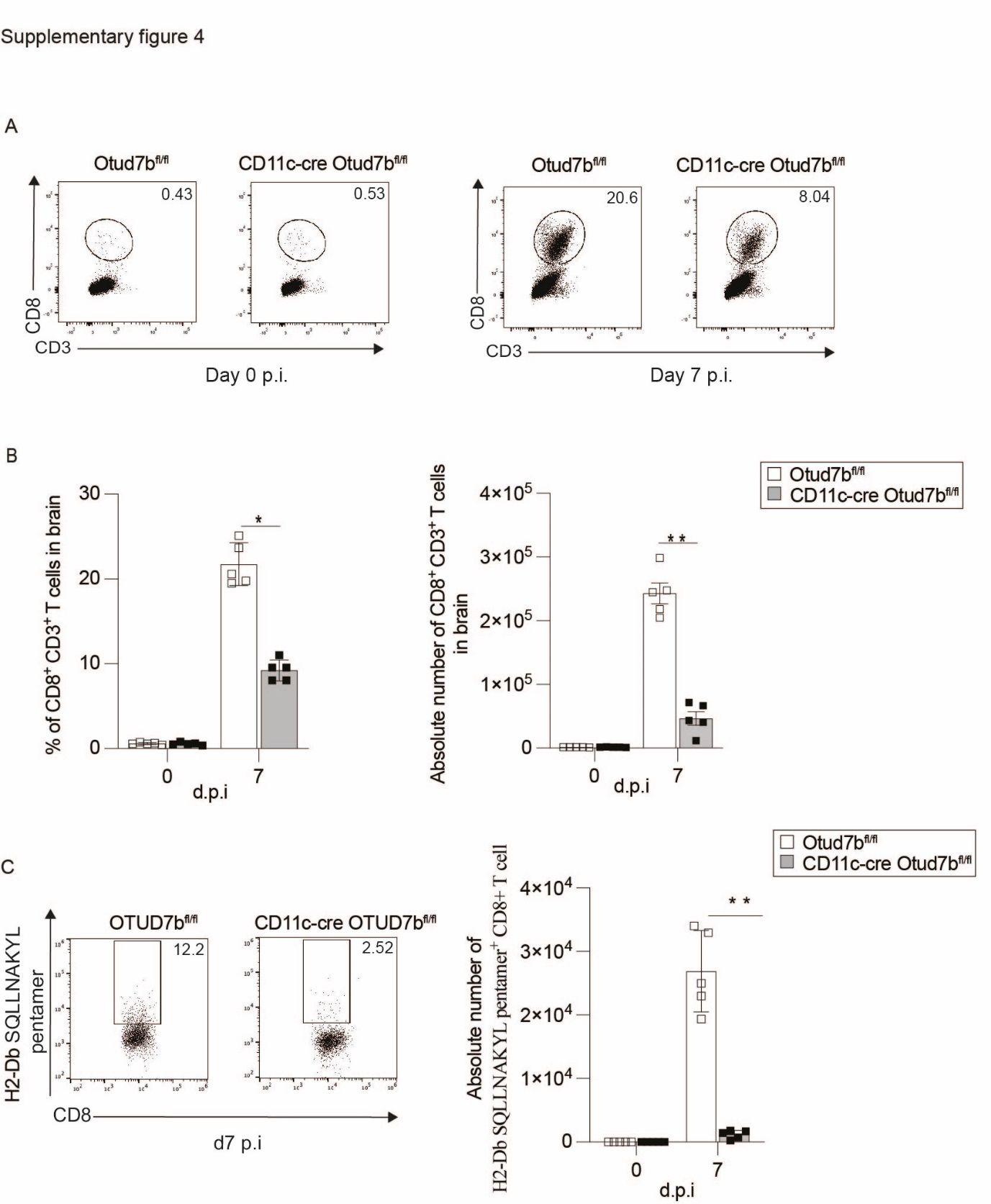


Supplementary Figure 4: Reduced recruitment of CD8^+^ T cells to the brain of *PbA*-infected CD11c-Cre Otud7b^fl/fl^ mice.

(A-D) Otud7b^fl/fl^ and CD11c-Cre Otud7b^fl/fl^ mice were infected i.p. with 1x10^6^ *Pb*A-infected RBCs. At day 7 p.i., mice were sacrificed and brains were harvested. (A) Representative dot plots showing the percentage of CD3^+^ CD8^+^ T cells in the brains of uninfected and infected (day 7 p.i.) mice (n = 5 per group). (B) Relative (left) and absolute (right) cell numbers of intracerebral CD3^+^ CD8^+^ T cells detected by flow cvytometry (n=5 per group). (C) Representative dot plots (left) of H2-D^b^ SQLLNAKYL pentamer^+^ CD8^+^ T cells in the brains of *Pb*A-infected mice at day 7 p.i. as determined by flow cytometry (n = 5 per group). Absolute numbers cell numbers of H2-D^b^ SQLLNAKYL pentamer^+^ CD8^+^ T cells (right) in uninfected (d0) and infected (day 7 p.i.) mice (n=5 per group). Data represent mean values + SEM. (B, D) Student’s t-test, *p<0.05, **p<0.01, p.i. post infection, RBC red blood cell.


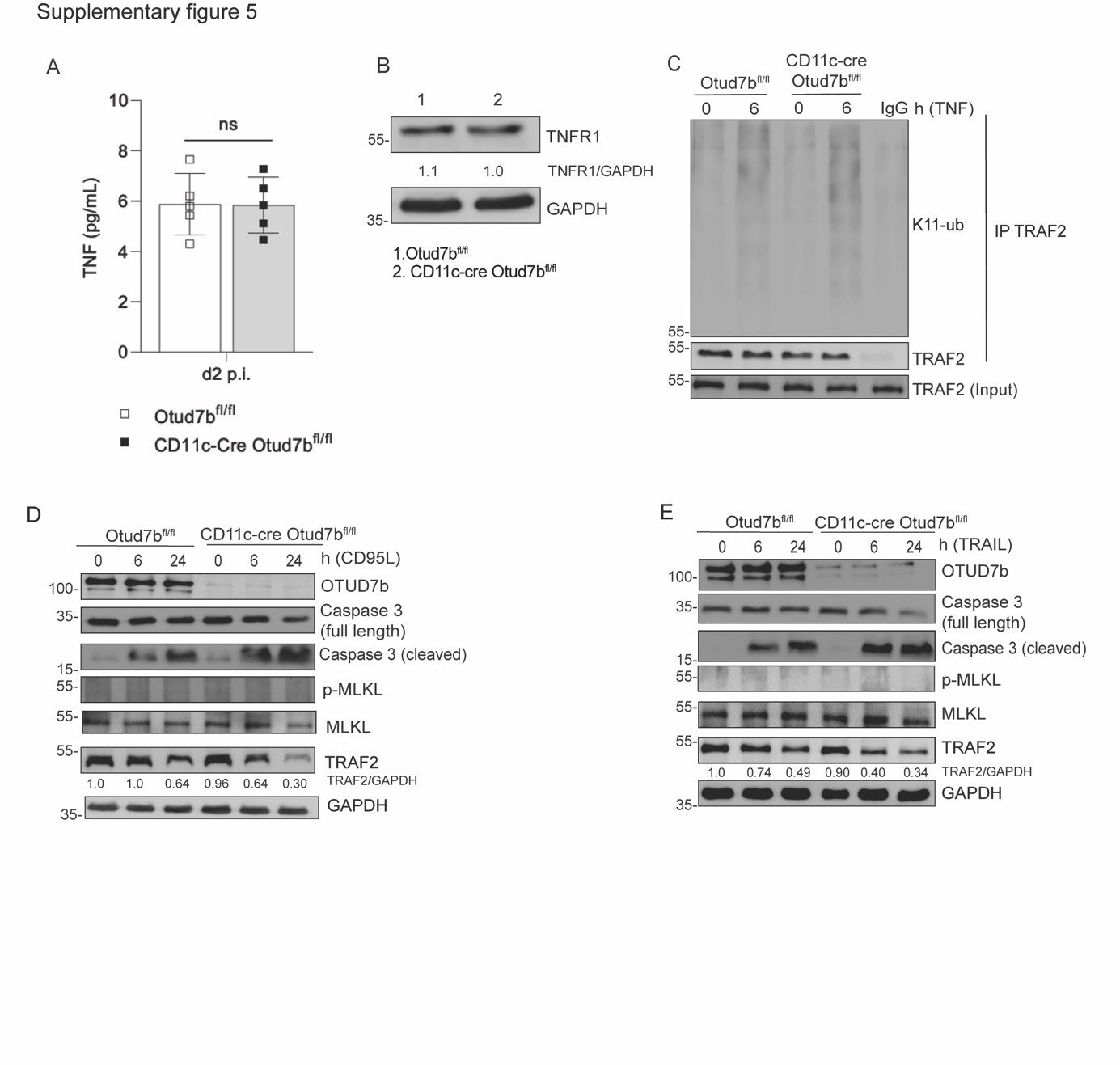


Supplementary Figure 5: DC- specific OTUD7b inhibits death receptor-mediated apoptosis

(A) TNF levels in serum of Otud7b^fl/fl^ and CD11c-Cre Otud7b^fl/fl^ mice were analyzed by CBA. (B) Untreated BMDCs of OTUD7b sufficient-and -deficient mice were analyzed by WB for TNFR1 expression. (C) Protein lysates of unstimulated and TNF stimulated (50ng/mL, 500U/mL) OTUD7b sufficient and -deficient BMDCs were immunoprecipitated with anti-TRAF2 antibody followed by WB analysis for K11-linked ubiquitination. (D,E) OTUD7b sufficient and -deficient BMDC were stimulated with 50 ng/mL (500U/mL) of CD95L (D) or (E) TRAIL. Cells were harvested after 6 and 24 h, respectively, and stained for the indicated proteins by WB. Data are representative for one of three independent experiments.
